# Bone Marrow Metastasis in Clear Cell Renal Cell Carcinoma: A Case Study

**DOI:** 10.7759/cureus.4181

**Published:** 2019-03-06

**Authors:** Samreen Khan, Sara A Awan, Sehreen Jahangir, Shawana Kamran, Imran N Ahmad

**Affiliations:** 1 Hematology, Shifa International Hospital, Islamabad, PAK; 2 Pathology, Shifa International Hospital, Islamabad , PAK

**Keywords:** renal cell carcinoma, clear cell renal cell carcinoma, bone metastasis

## Abstract

Clear cell renal cell carcinoma (RCC) is the most frequently reported renal cell neoplasm, which commonly metastasizes to the lungs, bones, lymph nodes, liver, adrenal gland and/or brain. It is usually diagnosed as an incidental finding on radiological imaging, which can further be confirmed by histological examination of the neoplastic tissue. Bone marrow metastasis of renal cell tumors is a rare event and very few cases have been reported. Here we report an unusual case of a 68-year-male who presented with lytic bone lesions on imaging. This raised the suspicion of a bone marrow involvement by a hematolymphoid malignancy or metastatic disease and a bone marrow biopsy was performed. Incidentally, the biopsy revealed infiltration of bone marrow by clear cell RCC. The patient was referred to the oncology clinic where further workup was done which revealed a primary renal tumor.

## Introduction

Renal cell carcinoma (RCC) constitutes 3% of all cancers worldwide [1]. It is further classified according to histology into clear cell RCC which accounts for 70% cases in adults, papillary RCC (10%-15%), chromophobe RCC (4%-6%), collecting duct carcinoma (less than 1%), and unclassified lesions(4%-5%) [2]. Most patients are elderly males who present between 60 and 70 years of age. As classic symptoms such as gross hematuria, pain and palpable abdominal mass are rarely seen, most affected individuals are diagnosed incidentally on abdominal imaging for other diseases. RCC is likely to metastasize to lungs, bones, lymph nodes, liver, adrenal gland and/or brain [1]. However, bone marrow metastasis is different from bone metastasis and according to literature, bone marrow metastasis precedes destruction of the normal bone architecture [3]. RCC presenting with bone marrow metastasis has rarely been reported. Here we report a case of an elderly male with clear cell RCC who presented with bone marrow metastasis of the tumor, confirmed on bone marrow biopsy.

## Case presentation

A 68-year-old male presented to our hospital with complaints of weight loss, fatigue and a progressively increasing mass over the left mandibular area for the past three months. On examination, the mandibular mass was firm, with no overlying skin changes or discharge. His past medical history included type two diabetes mellitus and chronic pancreatitis diagnosed eight months ago on computed tomography (CT) of the abdomen and pelvis from an outside institution. The CT also reported a 1.4 cm mass in the left kidney. Since his renal function tests were normal and there were no systemic complaints, no further investigations were ordered. Three months later the patient noticed bilateral swelling in armpits which were identified as bilateral axillary lymphadenopathy. Fine needle aspiration cytology of the left axillary lymph node revealed chronic lymphadenitis. Consequently, the patient was prescribed antibiotics. As the axillary lymphadenopathy persisted and the patient noticed new onset cervical lymphadenopathy, an otorhinolaryngology consultation was sought and CT of the neck was performed. The CT revealed bilateral cervical lymphadenopathy and small lytic lesions in the scapula, humerus, upper ribs and cervical vertebrae. This raised the suspicion of bone marrow involvement with a differential diagnosis of a lymphoma, multiple myeloma or metastatic disease. The patient's laboratory investigations on presentation are shown in Table 1.

**Table 1:** Laboratory parameters on admission

Test	Result	Normal Reference Range
Hemoglobin	15.8 g/dL	14-18 g/dL
White blood cells count	8070/μL	4000-11000/μL
Neutrophils	60%	50-70%
Lymphocytes	29%	20-40%
Monocytes	09%	2-10%
Eosinophils	02%	0-6%
Platelets count	280000 /μL	150000-450000 /μL
Reticulocyte count	1.1%	0.5-2.5%
Creatinine	1.51 mg/dL	0.72-1.25 mg/dL
Aspartate aminotransaminase	22 U/L	5-34 U/L
Alanine aminotransaminase	26 U/L	0-55 U/L
Alkaline Phosphatase	108 U/L	40-150 U/L
Total Bilirubin	0.6 mg/dL	0.2-1.2 mg/dL
Direct Bilirubin	0.295 mg/dL	0-0.5 mg/dL
Gamma-glutamyl transferase	227 U/L	0-64 U/L
Lactate dehydrogenase	236 U/L	125-243 U/L
Calcium	10.5 mg/dL	8.4-10.2 mg/dL
25-Hydroxy Vitamin D	32.3 ng/ml	30-150 ng/ml

**Figure 1:**
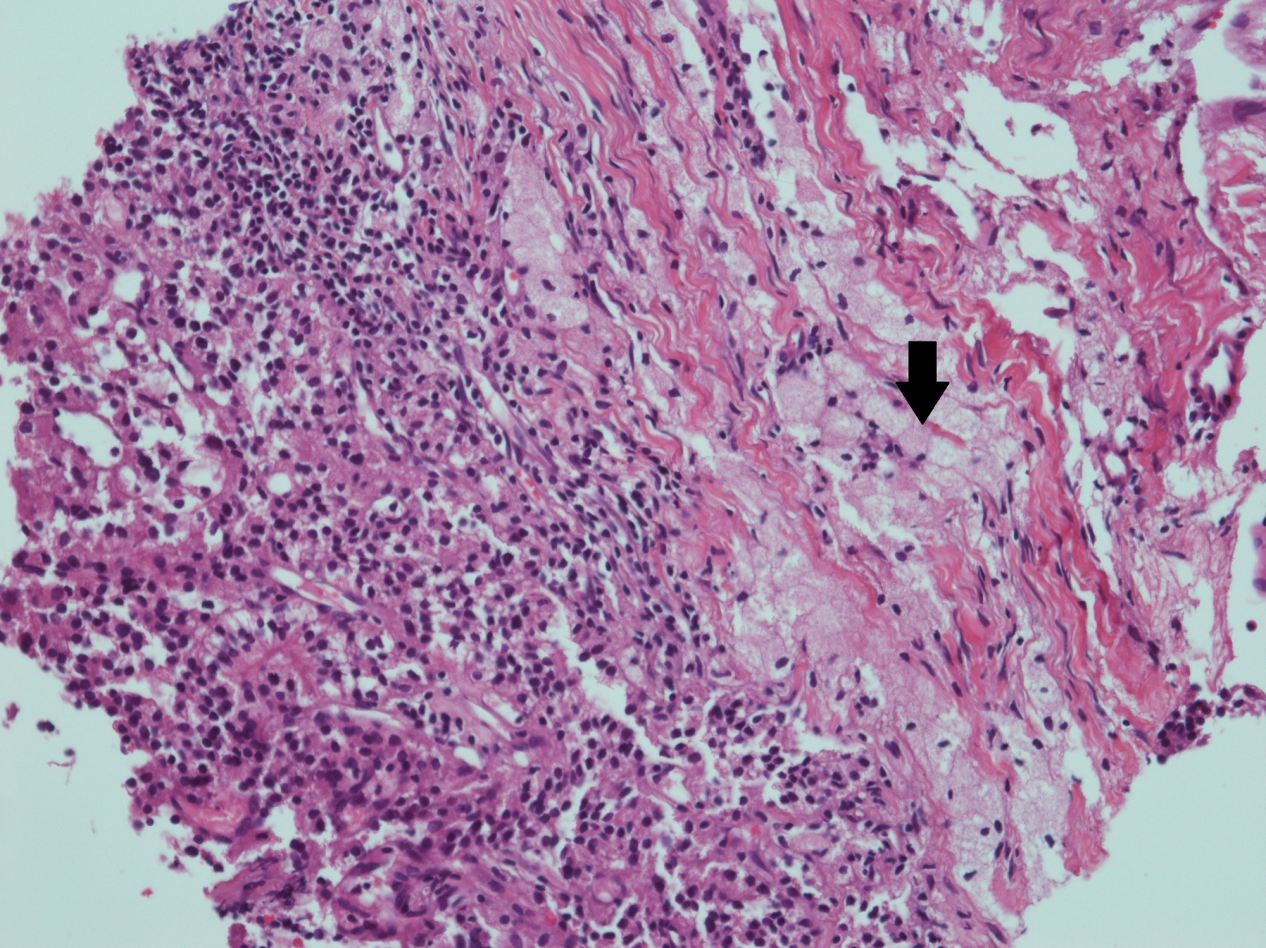
Tissue biopsy of the mandibular lesion Tissue biopsy shows a tumor consisting of polyclonal cells with abundant, clear cytoplasm and round to oval hyperchromatic nuclei.

A serum immunofixation electrophoresis was ordered which revealed normal levels of serum immunoglobulins G, A, and M, decreasing the likelihood of multiple myeloma. Tissue biopsy of the mandibular lesion exhibited a tumor comprised of nests of polygonal cells with abundant and clear cytoplasm. The nuclei were round to oval and hyperchromatic. A tissue biopsy from a lesion in the left rib revealed predominantly necrotic tissue with one fragment showing a tumor. The tumor comprised of atypical, ovoid cells with hyperchromatic nuclei and eosinophilic to clear cytoplasm. An admixed lymphocytic infiltrate was also seen. Figure 1 shows the tissue biopsy of the mandibular lesion.

**Figure 2:**
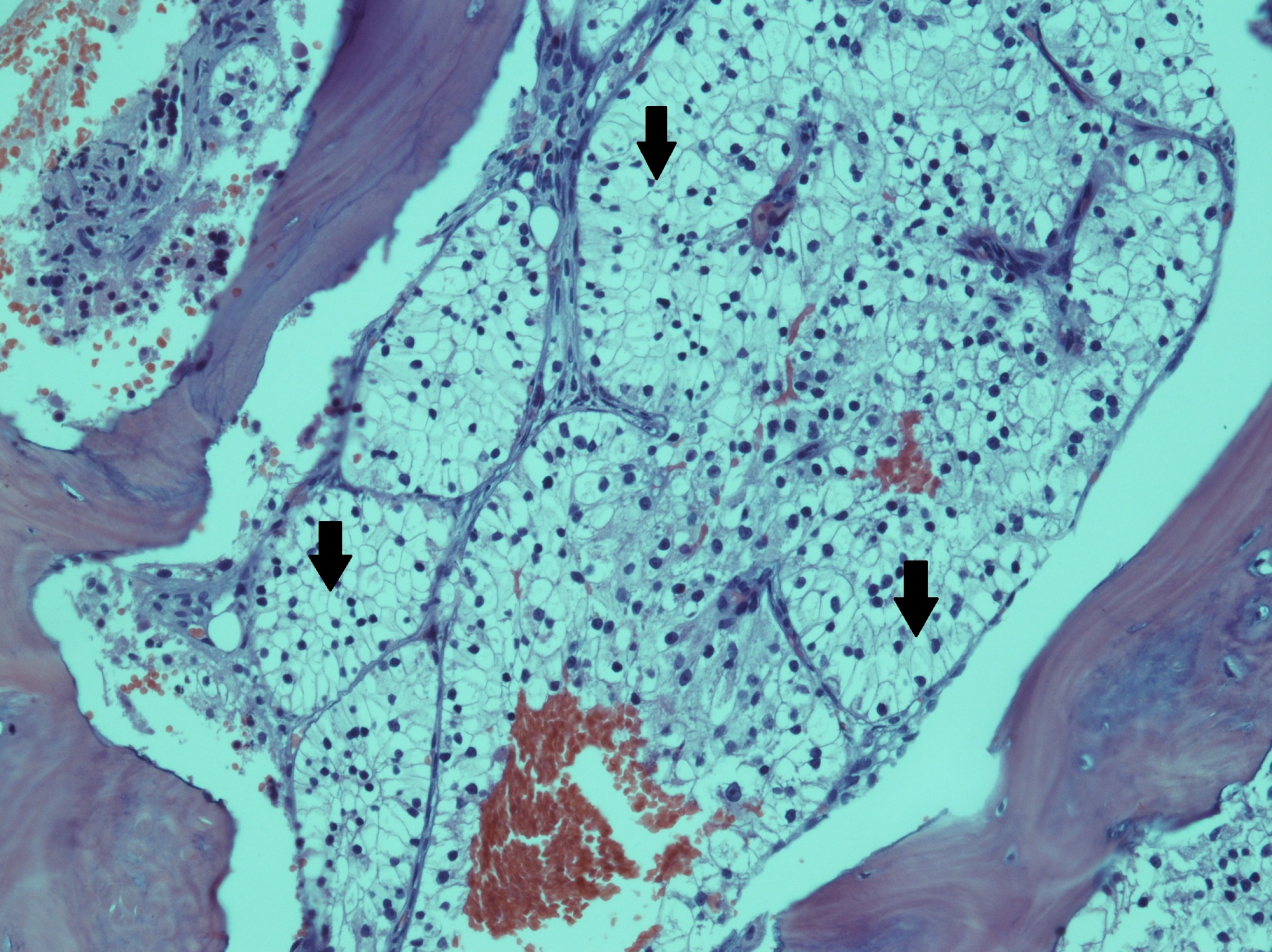
Bone marrow trephine biopsy Bone marrow trephine section showing an infiltrate composed of tubular structures lined by large cells with distinct borders and abundant, clear cytoplasm.

A bone marrow biopsy was performed to rule out bone marrow involvement. Bone marrow trephine revealed a hypercellular marrow showing infiltration by non-hematopoietic tissue, composed of tubular structures lined by large cells with abundant and clear cytoplasm. Figure 2 shows the bone marrow trephine biopsy.

**Figure 3:**
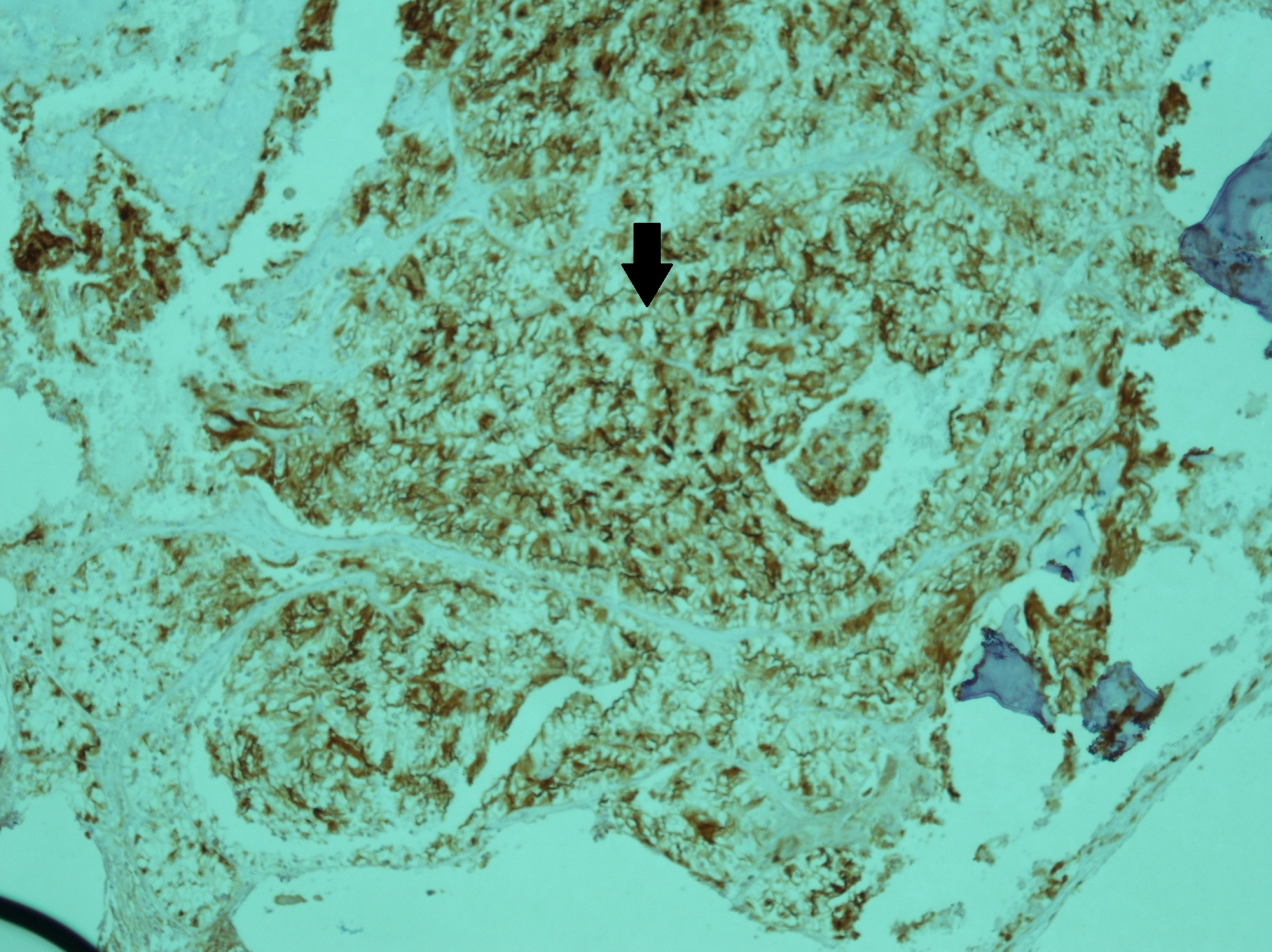
Bone marrow trephine section showing a CD10 positive infiltrate

The infiltrate was positive for cytokeratin AE1/AE3 and CD10 immunostains. CD10 is an immunostain that is found positive in proximal convoluted tubules. Figure 3 shows a section of the bone marrow showing positivity for CD10.

This confirmed the presence of metastatic renal carcinoma, likely clear cell RCC in the bone marrow. The patient was referred to the oncology clinic for further workup to identify the primary neoplasm.

## Discussion

Bone is a common site of metastasis for RCC, usually leading to osteolytic lesions. These lesions compromise the bone integrity and can cause skeletal-related events (SREs) including pain, fractures, nerve compressions and hypercalcemia [1]. A study conducted on a population of 803 patients with metastatic RCC being treated in a tertiary center revealed that 32% of patients presented with or later developed bone metastases [4]. In another study comprising of 11,157 patients of metastatic RCC, it was reported that 10% of patients with exclusive abdominal metastases also had bone involvement. The incidence was even higher (49%) in patients with abdominal, thoracic and brain metastases [5].

A multicenter study was conducted in Italy to determine the prognosis of patients who presented with metastatic RCC with bone involvement. Records of more than 1,800 patients who died from RCC from 1986 to 2011 were scrutinized, and 398 patients with bone metastases were identified. Majority of the patients (72%) were male and had a median age of 63 years [6]. Our patient was also an elderly male of 68, consistent with the demographic data of RCC patients reported worldwide. The study in Italy also observed that 71% percent of patients experienced at least one SRE. The median time to first, second, and third SRE were two, five, and twelve months, respectively [6]. However, although our patient had multiple lytic bone lesions, no SRE was reported.

Circulating tumor cells from solid organs can spread hematogenously and invade the highly vascularized bone marrow. Bone marrow involvement by a solid tumor can manifest as suppression of hematopoiesis causing anemia, thrombocytopenia and/or leukopenia. However, bone marrow metastasis can also exist without any changes in the hematologic parameters [7]. In our case report, the patient had normal blood counts and bone marrow biopsy was performed to rule out multiple myeloma in view of the lytic bone lesions.

Once the tumor cells seed into the bone marrow, it is followed by a dormant phase and a more aggressive active phase, both of which are regulated by multiple factors present in the microenvironment of the bone marrow stroma. Therefore, it can be said that bone marrow metastasis precedes architectural derangement of the bones which are detected much later by radiological imaging. This warrants the need to detect and preferably quantify bone marrow metastases as early as possible. An early diagnosis can lead to a better prognosis of the disease by effective management [3]. In a retrospective study, it was demonstrated that bone marrow metastases are an early form of skeletal metastases in breast cancer, and therefore early systemic treatment may preclude the development of bone metastases [8]. Bone marrow biopsy is a widely used method for diagnosis, staging and determining the prognosis of hematologic diseases. It can also be used to assess bone marrow metastasis in patients with solid tumors.

While bone marrow involvement in hematological malignancies is common, it is a relatively uncommon event in solid tumors. A study published in 2009 retrospectively analyzed 10,112 bone marrow specimens to analyze the frequency of bone marrow involvement in clinically unknown nonhematological malignancies and the primary sites of the metastatic neoplasms. The results revealed 101 (1.0%) specimens showing bone marrow metastases by nonhematological tumors. The primary lesions were identified in 50 of the 101 biopsy positive cases. The most frequent solid tumor identified on bone marrow metastasis was lung cancer (n= 11) and gastric cancer (n=11), followed by breast carcinoma (n=9) and prostate carcinoma (n=5). Only two cases showed renal carcinoma [9]. Schmid et al. evaluated 1,268 bone marrow biopsies obtained from 1,068 patients. Amongst these 95 patients (17.4%) with epithelial tumors showed bone marrow metastasis. These included predominantly prostatic carcinoma (54.1%), followed by breast (26.5%), gastric (18.5%) and lung cancer (7.9%) [10].

In a study conducted in Turkey, 3,345 bone marrow biopsies were examined. Fifty-eight patients were diagnosed with bone marrow metastasis of solid tumors including 39.7% with breast cancer, 32.8% had primary unknown tumor, 10.3% had gastric cancer, 6.9% had prostate cancer, 5.2% had lung cancer and only 1.7% had renal cancer. Among these 58 metastatic cases, 39 (67.2%) had a primary diagnosis before bone marrow biopsy was done. However similar to our case, 19 patients (32.8%) were firstly diagnosed from bone marrow biopsies as metastatic carcinomas [11].

A study was conducted by Jonsson et al. on 152 patients with different tumor types, to ascertain the usefulness of bone marrow aspiration for diagnosis of bone marrow metastases and to correlate morphology of aspirated tumor cells with the site of their origin. In this study, only seven cases of renal carcinoma were studied amongst which only one showed bone marrow metastases. The study observed the highest incidence of bone marrow metastasis in prostrate carcinoma and neuroblastoma patients [12]. Similarly, a study conducted at a cancer hospital in India described 90 bone marrow procedures done in cases of nonhematologic malignancies for suspected bone marrow involvement. Sixteen out of 90 patients were positive for metastases with the most common malignancy to metastasize being malignant small round cell tumor (Ewing's sarcoma and rhabdomyosarcoma), followed by carcinoma of breast and prostate. Only one case showed bone marrow metastasis of clear cell RCC [13].

Bone marrow metastasis in clear cell RCC is a rare event. However, performing a bone marrow biopsy in suspected cases of the aforementioned can help in timely diagnosis and management of bone marrow involvement.

## Conclusions

Bone marrow biopsy is a high yield test to identify bone marrow metastases of solid tumors. In some cases where the primary tumor presentation is occult, bone marrow examination with the application of immunohistochemistry on trephine sections can help determine the unknown primary tumor. This can help in identifying the disease in its early stages, thereby improving chances of survival.
